# Allergy to Mammalian Meat Linked to Alpha-Gal Syndrome Potentially After Tick Bite in the Amazon: A Case Series

**DOI:** 10.4269/ajtmh.20-1630

**Published:** 2021-09-20

**Authors:** Loïc Epelboin, Florent Roche, Maryvonne Dueymes, Geneviève Guillot, Olivier Duron, Mathieu Nacher, Félix Djossou, Angèle Soria

**Affiliations:** ^1^ Infectious and Tropical Diseases Department, Centre Hospitalier Andrée Rosemon, Cayenne, French Guiana;; ^2^ Equipe EA 3593, Ecosystèmes Amazoniens et Pathologie Tropicale, Université de la Guyane, Cayenne, French Guiana;; ^3^ Centre d’Investigation Clinique, INSERM 1424, Centre Hospitalier Andrée Rosemon, Cayenne, French Guiana;; ^4^ Université des Antilles et de la Guyane, Faculté de Médecine Hyacinthe Basturaud, Pointe-à-Pitre, France;; ^5^ Laboratory of Medical Biology, Centre Hospitalier Andrée Rosemon, Cayenne, French Guiana;; ^6^ Department of Pneumology and Gastroenterology, Centre Hospitalier Andrée Rosemon, Cayenne, French Guiana;; ^7^ Maladies Infectieuses et Vecteurs: Écologie, Génétique, Évolution et Contrôle, Centre National de la Recherche Scientifique, Institut pour la Recherche et le Développement, Université de Montpellier, Montpellier, France;; ^8^ Tenon Hospital, Dermatology-Allergology Department, Sorbonne University, Paris, France;; ^9^ Immunology and Infectious Diseases Center-Paris-INSERM U1135, Paris, France

## Abstract

The past decade has seen the emergence of a new type of food allergy occurring after ingestion of mammalian meat. This allergy is related to immunoglobulin (Ig)E specific for galactose-alpha-1,3 galactose (α-Gal). Originally described in the United States in 2009, other cases have subsequently been described in Australia and in Europe, but still very few in Latin America. The purpose of this study was to show the existence of this pathology in French Guiana and to describe the historical, clinical, and biological characteristics of these patients. Patients reporting an allergy to mammalian meat were included between September 2017 and August 2019. Eleven patients were included, nine of whom exhibited digestive symptoms; four, urticaria reactions; three, respiratory reactions; and five angioedema. The time between ingestion of red meat and reaction varied between 1.5 and 6 hours. The implicated meats were most often beef and pork. All patients had been regularly exposed to tick bites before the appearance of symptoms. All the samples (*n* = 7) were positive for anti-α-Gal anti-mammalian meats IgE. All the patients were Caucasian French expatriates. This study confirms the presence of this new entity in French Guiana and is the largest reported in Latin America. Our results do not clearly allow us to state that tick bites are the cause of this allergy, but all patients reported being exposed regularly to these arthropods.

## INTRODUCTION

The past decade has seen the emergence of a new type of food allergy characterized by various, sometimes severe (anaphylactic shock), reactions after ingestion of mammalian meat. This allergy is related to immunoglobulin (Ig)E specific to α-Gal the abbreviation of galactose-alpha-1,3 galactose an oligosaccharide. This oligosaccharide was identified in patients treated with cetuximab in the United States, a chimeric monoclonal antibody directed against the epidermal growth factor receptor, which is used in the treatment of colorectal cancers or squamous cell cancers of the head and of the neck.^1^ In the 2000s, several patients developed immediate hypersensitivity reactions, sometimes severe. The prevalence of these reactions was more than 20 times greater in the southeastern regions of the United States compared with the northeastern regions.^1^^,^^2^ These patients presented hypersensitivity reactions during the first cetuximab infusion. Indeed, they had developed IgE typical of this syndrome before exposure, specific for an oligosaccharide present on the Fab part of the heavy chain of the cetuximab: galactose-alpha-1,3 galactose (α-Gal).^2^^,^^3^ After the identification of this allergen, Commins et al. demonstrated the presence of IgE for α-Gal in patients who reported anaphylaxis reactions after consumption of beef.^4^ These reactions occurred late compared with traditional food allergy—3 to 6 hours after eating beef, pork, or lamb.^5^ An environmental sensitizing factor before exposure was suspected by the particular geographic distribution of these hypersensitivity reactions. Tick bites were hypothesized as a primary sensitizing factor because these reactions occurred in the same geographic area as Rocky Mountain spotted fever, which corresponds to the endemic area of the lone star tick (*Amblyomma americanum*) in North America. In addition, they found a 20-fold or greater increase in anti-α-Gal IgE levels in three subjects followed prospectively after episodes of multiple tick bites.^6^ At the same time, an Australian team was the first to discover the association between ticks bites and red meat allergy, describing a series of patients developing a delayed allergy to red meat secondary to tick bites, supporting this hypothesis.^7^ Subsequently, a German team also demonstrated that a population highly exposed to tick bites (forest service employees) had a high prevalence of IgE positivity for α-Gal.^8^ Since these findings, first reported in 2009, several other cases and case series have been reported, notably in Europe, Africa, and Asia^9^^–^^15^ (Table 1). In Latin America, only six cases have been reported, all during congresses but not published: four in Panama, one in Brazil, and one possible case in Costa Rica.^16^

**Table 1:** Review of the literature on described cases of α-Gal allergy

First author	Ref.	Year	Country	No. of cases	Delay before symptom onset (hours)	Symptoms	Meat	Anti-α-Gal IgE	Tick exposure
S. P. Commins	^5^	2009	USA	24	2–6	A, U, E	B, RM	1.9–100	UK
V. Nunen	^7^	2009	Australia	25	4	U, E, DR	RM	UK	*I. holocyclus*
S. Jacquenet	^30^	2009	France	2	1.5–4	A, U	P, B, C, L, M	0.28–4.82	UK
S. P. Commins	^6^	2011	USA	3	3–4	A, U	RM		*A. americanum*
R. Nunez	^10^	2011	Spain	5	3–5	A	B, P	9.7–100	*I. ricinus*
S. E. Wolver	^49^	2012	USA	3	4–6	Pr, A, Ap	B	3.94–100	Yes
M. Morisset	^9^	2012	France	14	2	A	B, P, Gi	0.4–294	UK
K. Sekiya	^15^	2012	Japan	1	5	A	B, P	UK	Yes
J. L. Kennedy	^50^	2013	USA	45	4	A, U, E	B, P, M	Positive	Yes
D. G. Ebo	^51^	2013	Belgium	9	1–6	U, E,	B, P M, ± milk	1.13–100	Yes
C. Hamsten	^11^	2013	Sweden	39	3–6	A, U	B, P	1.3–88	*I. ricinus*
M. F. Wuerdeman	^52^	2014	USA	1	5–8	U, Pa, Pr	RM	6.8	*A. americanum*
J. Fischer	^12^	2014	Germany	25	0.5–8	A, U, E	Gi, G, Milk	1.2–84	UK
A. M. Calamari	^13^	2015	Italy	1	6	A	RM	100–> 44	Yes
A. Guillier	^31^	2015	France	1	4	U, Pa, Ap, V	B, Gi	77	*I. ricinus*
E. Beaudouin	^32^	2015	France	1	4–5	U, V	Gi, milk, G	293	UK
C. Chatain	^33^	2015	France	2	5	UK	RM	1.27–100	No (wasps)
J. Fischer	^53^	2016	Germany	55	1–3	A	B, P, Ag, ab	1.22 − 100	UK
M. Kaloga	^14^	2016	Ivory Coast	1	3	Ap, Pr	B, M, Ag	11.1	UK
R. R. Cocco	^16^	2016	Brazil	1	6	U, Pr	B, P, M	70.7	Yes
D. Villalta	^54^	2017	Italy	40	2–4	U, V, Ap, E	B, M, P, Milk, G	100	Yes
A. J. Bircher	^55^	2017	Switzerland	4	2–3	A, U, Ap	RM	1.26–71	Yes pour 1
G. Arslan Lied	^37^	2017	Norway	1	6	Pr, U	B, P, M	100	Yes
C. Abreu	^56^	2018	Portugal	1	1	Ap, V, U	RM	35.3	UK
A. C. Kaplan	^57^	2018	USA	1	6	D, V, Pr, U	B	2.41	*A. americanum*
W. L. Jackson	(^58^	2018	USA	1	6	Ap, D, U, Pr	B, Milk	8.45	*A. americanum*
J. K. Khoury	^59^	2018	USA	3	UK	U, Ap	RM	14–49	*A. americanum*
T. Mabelane	^60^	2018	South Africa	84	0.75–6.25 (median 1.75)	U, Ap	RM	Median 4.2 (IQR 1.9–11.0)	UK
J. M. Wilson	^38^	2019	USA	245	2	U, A, D, Ap	RRM	Positive	UK
V. Pisazka	^61^	2019	Austria	1	8	U, Pr, V, D	B	100	Yes
A. Hodzic	^34^	2019	France	1	3–4	A, U, Ap	Barbecue	1.75	*I. ricinus*

*A *= *Amblyomma*; A = anaphylaxis; Ag = agouti; Ap = abdominal pain; B = beef; D = diarrhea; E = edema; G = gelatin; Gi = giblets; *I* = *Ixodes*; IQR = interquartile ratio; L = lamb; M = lamb; P = pork; Pr = pruritus; RM = red meat; U = urticaria; UK = unknown; V = vomits.

The mechanism by which the tick bite triggers the IgE antibodies to α-Gal is still poorly understood. Recent studies tend to show that this response is caused by α-Gal derived from ticks. Indeed, studies have shown the presence of α-Gal in the gastrointestinal tract of a European species, the castor bean tick *Ixodes ricinus*, and in the saliva of a South American species, *Amblyomma sculptum*.^17^^,^^18^ In addition, a team has demonstrated the endogenous synthesis of α-Gal for another North American species, the black legged tick *Ixodes scapularis*.^19^ It was recently shown that α-Gal is also present in tick cement and that tick gut microbiota contains α-1,3-galactosyltransferase genes that potentially contribute to α-Gal content in tick guts and salivary glands.^20^^,^^21^ Three main hypotheses have been proposed to explain the source of the glycan that leads to an IgE response directed to the α-Gal epitope.^1^ Certain ticks could intrinsically produce α-Gal, either at baseline or during feeding^2^; the α-Gal present in tick saliva would be residual from a prior blood meal of a nonprimate mammal,^3^ and the α-Gal present in tick saliva could be produced by Rickettsiales or another microbial symbiont of ticks.^22^

However, it is not yet known what mechanisms are behind the production of IgE antibodies to α-Gal.^23^ Although present in abundance in most mammals, it is absent in humans and nonhuman primates of the Old World. In humans, the B antigen present in red blood cells has structural similarity to α-Gal and appears to be a protective factor for α-Gal-mediated red meat allergy.^24^^–^^26^ α-Gal is also absent in other nonmammalian vertebrates, such as birds, reptiles, and fish.

French Guiana is a French territory located in the northeastern part of South America in the Amazon region, which has important ethnic and cultural diversity. It also has important biological diversity, with 90% of the territory covered by equatorial forest. Many tick species are present, exposing the population to potential bites. A study published in early 2019 listed 22 species of ticks in French Guiana, half of which had been collected from humans. The two most abundant species were *Amblyomma cajennense* and, to a lesser extent, *A. oblongoguttatum*, both commonly found in humans.^27^ These two species of ticks belong to the same genus and are part of the Ixodidae, the family of hard ticks, like *Amblyomma americanum* (which, however, has not been found in French Guiana). An older study from 2006 also listed 28 species of Ixodidae that had been found in humans in South America, of which three were found frequently, with *Amblyomna ovale* in addition to the two previously mentioned.^28^

The objective of the study was to show the presence of mammalian meat allergy in French Guiana and describe its sociodemographic, clinical, and biological determinants.

## METHODS

### Study type and study population.

A prospective observational and descriptive study was carried out between September 2017 and July 2019 in French Guiana on patients reporting a suspicion of allergy after ingestion of mammalian meats. All patients reporting a suspicion of food allergy after ingestion of mammalian meat were prospectively included. A call for cases via medical and nonmedical networks took place between September 2017 and July 2019. Patients were recruited through several routes. Some had already consulted in the Department of Infectious and Tropical Diseases for another illness or were identified by the investigators beforehand through word of mouth. Others were found through a mailing to the general physicians of the territory (≈105 people) and to practitioners at the Cayenne Hospital Center (≈205 people) asking if they had dealt with a patient complaining of an allergy to red meat. Still others were recruited via nonmedical networks, such as mailing lists to naturalist networks (ornithologists, mammologists, chiropterologists, and herpetologists: ornithoguyane@yahoo.fr [≈261 people], herpetoguyane@yahoo.fr [≈93 people], chiroguyane@yahoo.fr [≈63 people]), as well as the Facebook group LVG—la vie en Guyane (Life in French Guiana, posted on August 4, 2019), which reached more than 31,000 people living mainly in French Guiana and coming from all ways of life and ethnicities.

### Inclusion and exclusion criteria and case definition.

Patients reporting a suspicion of repeated allergic reactions after ingestion of mammalian meat were included. The inclusion criteria were a compatible clinical picture characterized by allergic (cutaneous, mucous, respiratory, or digestive) reactions after ingestion of any mammalian meat and the appearance of the first signs in French Guiana.

The exclusion criteria were patients with no follow-up and thus without medical records, clinical history not consistent with the diagnosis, and first signs of reaction having started outside of French Guiana.

Proven cases were individuals reporting clinical symptoms consistent with the α-Gal syndrome clinical picture described in the literature and confirmed by paraclinical examinations. The probable cases were individuals reporting compatible clinical symptoms without laboratory results.

### Data collection.

Patients were seen in a dedicated consultation in the infectious and tropical diseases department of Cayenne Hospital and answered a standardized questionnaire after signing informed consent forms.

The questionnaire included the following variables: self-assessment of reactions occurring after consumption of red meat, history of allergy, exposure to ticks, and lifestyle. Because patients had difficulty estimating the date of their first bites, the date of arrival in French Guiana was taken into account to estimate the time between tick bites and the onset of the first allergic symptoms.

The patients were then explored with standard biology, and a blood sample was taken either by the laboratory of the Cayenne Hospital Center or in a private laboratory, and the analysis of specific IgE was performed at Biomnis laboratory in Lyon.

### Statistical analysis.

Continuous variables were analyzed to obtain the median, interquartile range (IQR), and range. Categorical variables were described by percentages.

### Determination of anti-α-Gal IgE.

Specific IgE antibodies to α-Gal and to the most common mammalian meats (beef, pork, lamb), but also fish, chicken, and duck and cow and goat milk (depending on patient history). Patients were free to choose the laboratory where they carried out their analyses. Most allergological assays were carried out by the Eurofins Biomnis laboratory, France. Samples were generally measured using the commercially available ImmunoCAP assay (Thermo Fisher Scientific/Phadia, Hycor, Waltham, MA), and results were expressed in kU/L. For the Thermo Fischer/Phadia technique, specific IgE can be quantified between 0.1 and 100 kU/L. The positive thresholds used by the laboratory are therefore IgE > 0.1 kU/L. The Hycor technique used to test IgE against duck meat had a positivity threshold of 0.35 kU/L. Total IgE was performed in the Cayenne Hospital laboratory and measured using the commercial test FEIA (Phadia, Thermo Fischer Scientific) in serum. The positive threshold was 150 kU/L. A ratio between specific IgE antibodies to α-Gal and total IgE was calculated for six patients and expressed as a percentage.

### Ethics.

The patients were informed of the study objectives, performed in routine care, and all signed a consent form. The data were anonymized for analysis. According to French legislation, local ethics committee accord is not necessary for an observational study.

## RESULTS

Between September 2017 and July 2019, 15 patients responded to the call for information; two patients did not follow up after the first contact, 2 patients had a clinical history that did not match the criteria (one patient had clinical manifestations episodically only during her pregnancy and one had clinical manifestations only with chicken meat). The demographic and historical characteristics of the patients are detailed in Table 2, the biological and historical continuous variables are described in Table 3, and the details of the allergological biological assessment are shown in Table 4. Eleven patients were included, with a male/female ratio of 0.46. All (11 of 11) patients were White from mainland France, with a median age of 38 years (IQR 33–39.5, range 30–54 years) (Tables 2 and 3). Specific patient details are reported in Table 5. Four patients declared preexisting lactose intolerance, three had local reactions to wasp stings, and one reported an allergy to penicillin. Only one patient (no. 2) has an atopic history with allergic asthma and atopic dermatitis. Nine patients were aware of their ABO grouping (six group A, three group O, and none were group B). Skin prick tests with mammalian meats were not performed in our study because they were not available; however, only one patient (no. 2) had negative pork skin prick.

**Table 2:** Demographic and historical characteristics of the 11 patients (categorical variables)

	Outcome	Number (*N* = 1)	Percentage
Gender	Male	5/11	45
	Female	6/11	54
Age categories (years)	0–30	1/11	9
	31–60	10/11	91
	> 60	0/11	0
Geographic origin	Mainland France	11/11	100
	Other	0/11	0
Symptoms	Skin manifestations	8/11	73
	Urticaria	4/11	36
	Angioedema	5/11	45
	Respiratory manifestations	4/11	27
	Dyspnea	3/11	27
	Severe angioedema	1/11	9
	Digestive manifestations	9/11	82
	Abdominal pain	7/11	64
	Vomiting	5/11	45
	Diarrhea	5/11	45
Type of meat consumed (patient reported)	Beef	10/11	91
	Pork	9/11	82
	Rabbit	2/11	18
	Lamb	3/11	27
	Milk/dairy products	2/11	18
Risk factor	Tick exposure	11/11	100
Blood group	A	6/9	67
	O	3/9	33
Symptoms present at time of investigation	Yes	5/11	45
	No	3/11	27
	Unsure (no longer eats meat)	3/11	27

**Table 3:** Historical and laboratory characteristics of the 11 patients except for α-Gal IgE level (seven patients) (Continuous variables)

Variables	Median	Interquartile range 25–75	Range
Age (years)	38	33–39.5	30–54
Time between arrival in French Guiana and onset of symptoms (years)	3	2.5–9	1–11
Time between ingestion of meat and onset of symptoms (hours)	3	2 h 15 min– 5 h 30 min	1 h 30 min– 6 h
α-Gal IgE level (kU/L)	6.5	5.5–11	1.7–56

**Table 4:** Allergological Assessment of seven patients

Patient	2	4	6	7	8	9	10
Total IgE levels (kU/L)	2,170*	**245**	**76**	**437**	**518**	**233**	
α-Gal IgE levels (kU/L)	**56**	**12.8**	**15.3**	**6.49**	**5.14**	**1.66**	**6.52**
α-Gal levels/total IgE levels ratio	3%	5%	20%	1.5%	1%	< 1%	
Beef IgE levels (kU/L)	**63.2**	**3.58**	**1**	**3.16**	**1.57**	**0.33**	**1.45**
Pork IgE levels (kU/L)	**58.6**	**2.41**	**0.7**	**2.56**	**0.87**	**0.17**	**0.89**
Lamb IgE levels (kU/L)			**0.72**	**1.91**			**0.29**
Rabbit IgE levels (kU/L)	**30.9**						
Chicken IgE levels (kU/L)	**2.23**		< 0.1	< 0.1	< 0.1	< 0.1	< 0.1
Duck IgE levels (kU/L)	< 0.35		< 0.35	< 0.35	< 0.35	< 0.35	
Cow milk IgE levels (kU/L)	**29.2**		< 0.1	**1.47**	**0.35**	**0.12**	**0.18**
Goat milk IgE levels (kU/L)				**0.35**	**0.11**		

Ig = immunoglobulin. Bolded numbers represent positive results.

* Patient with an atopic site; therefore, elevation of specific IgE difficult to interpret.

**Table 5:** Summary of clinical data and demographic characteristics of each of the 11 patients

Case	Age (years)	Gender	Skin symptoms	Digestive symptoms	Resp. symptoms	Anaphyl. shock	Meat	Mean delay (hours)	Living area	Time in forest	Job	Tick exp.
1	39	Male	Urticaria, angioedema, erythema	Diarrhea, vomitus, abdominal pain	Dyspnea	Yes	Beef, pork, lamb	6	Urban	+++	Ranger	Yes
2	38	Male		Abdominal pain	Dyspnea	No	Beef, pork, deer, rabbit	2.5	Rural	+++	Agr. advisor	Yes
3	36	Male		Abdominal pain		No	Beef, pork, rabbit	1.5	Urban/rural	+++	Trade	Yes
4	38	Female	Angioedema	Diarrhea, abdominal pain		No	Beef, pork	3	Urban	++	Water and sanitation technician	Yes
5	40	Male		Vomitus, abdominal pain		No	Beef, pork	1.5	Rural	+++	Translator	Yes
6	30	Female	Urticaria	Vomitus, abdominal pain		No	Beef	5	Urban	++	Trade	Yes
7	32	Female	Urticaria, angioedema			No	Beef, pork, lamb	3	Rural	+++	Teacher	Yes
8	54	Female		Diarrhea, vomits, abdominal pain		No	Beef, pork	6	Rural	++++	Writer	Yes
9	34	Female	Urticaria	Diarrhea, abdominal pain		No	Pork kidneys, dairy products	2	Urban	+++		Yes
10	44	Male	Angioedema	Diarrhea, vomits, abdominal pain		No	Beef	4	Urban/rural	+++	Teacher	Yes
11	32	Female	Angioedema		Dyspnea	No	Beef, pork, lamb	1.5	Rural	++	Driving school secretary	Yes

Agr. = agricultural; Anaphyl = anaphylactic; exp = exposure; Resp. = respiratory. Time in forest: ++++ = lives in forest; +++ = very frequent trips; ++ = regular trips in forest.

The 11 patients included reported skin, respiratory, and/or digestive manifestations some hours after mammalian meat ingestion (Table 2). Most of the patients reported, abdominal pain, (*n* = 9, 82%), associated with diarrhea or vomiting for seven of them. Four of these 7 patients also reported skin symptoms, such as urticaria (3 patients) or angioedema (3 patients). One patient (no. 9) had only skin symptoms with hives and angioedema, and three patients had respiratory symptoms, such as dyspnea with hypoxemia requiring corticosteroid treatment.

Patient 9 was admitted in the emergency department after anaphylactic shock with abdominal pain, diarrhea, trunk erythema, tachycardia, and hypotension (70/50 mm Hg), 7 hours after ingesting a beef burger. The episode resolved after injection of adrenaline and systemic steroids. Fifteen days earlier, he had a similar reaction several hours after ingesting ground beef. One month earlier, he had suffered an angioedema after a wasp bite.

Patients reported a delay between mammalian meat consummation and clinical manifestations varying between 1.5 and 6 hours with a median at 3 hours (IQR 2.25–5.5 hours). The symptoms lasted between 7 and 24 hours. Symptom onset began after the arrival of patients in French Guiana, with a median delay of 3 years (IQR 2.5–9.0; min–max 1–11 years). Two-thirds of the patients (7 of 11) included reported eating beef or pork before the onset of symptoms. Concerning the mammalian meats ingested, two patients report reactions only after ingesting beef, one after pork offal, and two others each after lamb or rabbit. Only one patient (no. 8) reacting with pork also reported abdominal discomfort after consumption of dairy products. After avoiding mammalian meats in their diet, all patients reported symptom resolution. In addition, three patients reported being able to eat small amounts of mammalian meats (beef and pork) again after following an avoidance diet.

All patients reported frequent journeys in the Amazon rainforest, an area with high exposure to ticks in Guiana, about half (six of 11) also lived in rural areas. All patients reported that they were regularly bitten by many ticks. For all the patients, there was a temporal link between the onset of tick bites and the onset of allergic reactions, which began after numerous tick bites.

Of the 11 patients, seven were benefitted from a serum analysis with a specific IgE assay (Table 4). All were found to be positive for α-Gal (median 6.5 kU/L; IQR 5.5–11.2; range 1.7–56). All the sera analyzed (*n* = 7) had positive IgE to beef and pork. The three patients who received a sheep-specific IgE assay were positive (0.29–1.91 kU/L). All sera analyzed for duck-specific IgE (*n* = 5) were negative, including the patient with chicken-specific IgE.

## DISCUSSION

Here we report a series of 11 cases of patients with an allergy to mammalian meat, mainly beef, pork, and lamb, acquired in French Guiana, seven of whom had concordant biological results.^22^^,^^29^ This is, to our knowledge, the largest series in Latin America where only six cases had been previously described.^16^ α-Gal remains rare and unique because it does not have the usual chronology of food allergies and is an allergy to a sugar, whereas all other food allergies are to proteins. Allergy to α-Gal is a little-known pathology of recent description, dating back to 2009, and although an increasing number of cases are reported, most observations available in the literature are case reports or small series, mostly from the United States.^23^ In France, only about 20 cases have been described.^9^^,^^30^^–^^34^

There were two main classes of symptoms in patients with an allergy to “red meat”: symptoms of the purely anaphylactic series and digestive symptoms. These symptoms generally appear several hours after a meal, which can make it difficult to identify the responsible food. A U.S. study showed that the average time between symptom onset and diagnosis of mammalian meat allergy for 80% of patients was 7.1 years, and the remaining 20% were diagnosed within 1 year.^35^ In our series, digestive symptoms were preponderant and included abdominal pain, vomiting, and diarrhea, either isolated or associated with skin signs such as urticaria or angioedema. The literature reporting large series shows a higher prevalence for digestive manifestations, isolated or not,^34^^,^^36^ as found in our study.

There was significant variability in the quantification of specific IgE for the patients who could benefit from the assay, with anti-α-Gal IgE varying between 1.66 kU/L and 56 kU/L with no correlation with the severity of symptoms. Cases reported in the literature also support this, with large variations in anti-α-Gal IgE levels.^34^^,^^37^ A US study on a large cohort of 245 patients found the same results and found that patients with isolated digestive symptoms had relatively lower anti-α-Gal IgE titers.^38^

Classically it has been shown that allergy to mammalian meat is characterized by a delayed onset of allergic symptoms, between 3 and 6 hours after ingestion of the food. In our study, four subjects with positive titers for IgE α-Gal had earlier reactions (from 1.5 to 2.5 hours) showing that the delay in the allergic reaction is not systematic. This has already been reported in the literature.^5^^,^^9^^,^^12^ There are reports that associate reactions with the amount of α-Gal present in the food (pork kidney rich in α-Gal), the presence of cofactors such as alcohol, nonsteroid anti-inflammatory drugs, exercise, sex hormones, or other biological differences between men and women.

Finally, in accordance with the data available in the literature, our cohort did not include any individual from the type B blood group, which is in line with the protective nature of group B, even in our small sample. The frequency of blood group B is low in the United States and in Europe,^39^ and very rare in afrodescendents, which could affect the interpretation of our results; however, 100% of the patients of our study were White Caucasians from mainland France. This is surprising because White people from mainland France (called “metropolitans”) represent only ≈10% of the ethnically diverse population of French Guiana. There is no doubt that all ethnic groups are exposed to the forest and tick bites in French Guiana; Creoles, Brazilians, Maroons, Hmongs, others are among the people who work in the forest as loggers, gold diggers, forest rangers, hunters, and fishers, for example. One explanation would be recruitment bias due to the predominance of French Caucasian subjects, but the social networks used for enrollment were broad and reached many ethnic groups. Lack of knowledge of this pathology on the part of the medical community and the general population could have hampered the reporting of cases, as well as the low specificity of isolated digestive symptoms.

This study does not allow us to establish a clear causal link between tick bites and allergy to mammalian meat; however, all patients were regularly exposed to ticks, and all were bitten before the onset of symptoms, which occurred in French Guiana in delays that seemed compatible with a sensitization within the region. In addition, several studies show α-Gal sensitization after tick bite.^22^ There is also asymptomatic sensitization for α-Gal^40^; a German study found a prevalence of α-Gal sensitization > 0.35 Ku/L of 19.6% against only 8.6% actually allergic to mammalian meat in a population exposed to tick.^8^ Interestingly, studies suggest that this sensitization appears to decrease over time and increase again after additional bites,^41^ suggesting that recent or continued exposure to ticks is an important factor in sensitization to α-Gal. Ideally, this study would have included a control group of patients without red meat allergy with whom we could have performed the IgE anti-α-Gal^40^ assay, but this could not be done. As mentioned earlier, several tick species have been implicated around the world: *A. americanum* in North America, *I. ricinus* in Europe, and *I. holocyclus* and *I. australiensis* in Australia.^42^ Although in our study, no species of tick was formally identified, we know that many species are present in French Guiana,^27^ with a preponderance of the Cayenne tick *A. cajennense*, which has also been commonly found on humans. This species has also been implicated in studies identifying Panamanian cases^16^ and is closely related to the lone star tick *A. *americanum,^43^ which is associated with allergy to mammalian meat in North America.^5^ All identified tick species are part of the so-called hard tick family. In addition, in 2017, a Brazilian team showed that the injection of saliva of *A. sculptum*, a member of the *A. cajennense* species complex, in mice whose alpha 1–3 galactosyltrasferase was inactivated, produced IgG and IgE anti α-Gal, suggesting that this species of tick endemic to the Amazon could be a vector of this allergy.^18^ These findings suggest that French Guiana probably has at least one tick species that may be the source of this allergy. Interestingly, a recent publication showed that there would be a competition between anaphylaxis from fire ants and anaphylaxis with α-Gal, which would explain why the southern United States has both *A. americanum* and fire ants but a low rate of α-Gal anaphylaxis because fire ants are a predator of ticks. However, fire ants such as *Solenopsis* are common in French Guiana, and an equivalent phenomenon could be described.^44^^,^^45^

Ticks are vectors of many infectious bacterial diseases (Lyme disease, rickettsioses, ehrlichiosis), viral or parasitic. A study published in 2017 showed that sensitization to α-Gal was common in a tick-endemic area in Sweden but that there was no relationship with a history of Lyme borreliosis.^47^ Lyme disease is currently a hot topic in infectious pathology and a source of countless controversies both within the medical world and among scholarly societies, physicians, and patient associations.^50^ The diagnosis of Lyme disease is often complicated and sometimes established by default, with serological tests that can be negative leading to the prescription by some doctors, known as “Lyme doctors,” of heavy, often unnecessary antibiotic protocols, especially in patients with persistent symptoms after a well-conducted initial treatment. In 80% of these patients, a diagnosis other than Lyme borreliosis is made,^46^ and the latest recommendations from the French Language Infectious Pathology Society, published in 2019, recommend no further treatment after the initial treatment.^47^ These patients may present with polymorphic symptoms associating asthenia, joint pain, and digestive symptoms in the context of tick bites. These symptoms overlap with those of α-Gal allergy. Commins et al. used a mammalian meat-avoidance diet in these patients, several of whom saw remarkable improvement in symptoms or even resolution of chronic symptoms, leading to the hypothesis that many patients with persistent Lyme disease may in fact have an intolerance or even a clear allergy to α-Gal.^48^ The fact that the main vector of Lyme disease agent in Europe, the castor bean tick *I. ricinus*, was also shown to harbor α-Gal in its gastrointestinal tract,^17^ further reinforces this hypothesis.

The precise natural history of an α-Gal allergy in mammalian meat is not yet known. In our study, three patients subsequently reported to us that they were able to consume red meat without triggering an allergic reaction. Two of these patients had moved back to mainland France, hence less exposed to tick bites. However, we do not currently know the potential anaphylactic risk of such consumption, especially with co-factors. Thus, the current recommendations are strict meat avoidance and a threshold dose has never been shown. The other patients who no longer see allergic reactions continue with their avoidance regimen, making it difficult to assess the fading of reactions.

## CONCLUSION

This study is the first description of α-Gal syndrome due to mammalian meats in the Amazon region and the largest series ever reported in Latin America. With the comprehensive clinical description and biological results, it confirms the presence of this little-known disease on the continent. Because of its nonspecific symptoms and the delayed onset of reactions, α-Gal syndrome is probably a neglected disease on the continent. Although the mechanism of tick sensitization is still poorly understood, some studies are beginning to look at the proteomes of these organisms in Sweden, Japan, and Brazil.^23^ It would be interesting to study French Guiana ticks to confirm their role in α-Gal sensitization.
